# From Food Waste to Edible Packaging: Development and Characterization of Biodegradable Gelatin Films with Microfibrillated Cellulose from Cowpea Pod Skin (*Vigna unguiculata*) and Corn Straw (*Zea mays*)

**DOI:** 10.3390/foods14173033

**Published:** 2025-08-29

**Authors:** Priscila Santos Souza, Cristiani Viegas Brandão Grisi, Rita de Cassia Andrade Silva, Emanuel Marques da Silva, Fábio Anderson Pereira da Silva, Antonia Lucia de Souza

**Affiliations:** 1 Postgraduate Program in Chemistry, Federal University of Paraíba, João Pessoa 58051-900, Brazil; priscila.s_souza@hotmail.com (P.S.S.); ritaandrade112@gmail.com (R.d.C.A.S.); 2 Postgraduate Program in Food Science and Technology, Federal University of Paraíba, João Pessoa 58051-900, Brazil; emanuel.marques2015.2@gmail.com (E.M.d.S.); fabio.silva@academico.ufpb.br (F.A.P.d.S.)

**Keywords:** active packaging, microfibrillated cellulose, cowpea pods, corn straw, gelatin film

## Abstract

This research focused on the development and characterization of gelatin-based films incorporated with cellulose microfibrils (CMFs) extracted from cowpea pod skin (*Vigna unguiculata*, CPMC) and corn straw (*Zea mays*, CSMC). The use of CPMC to produce gelatin films has not been previously reported in the literature. Eleven formulations were prepared based on a 2^2^ factorial design with four axial and three central points, in addition to a control film (FC) composed of 1.00% gelatin and 1.00% glycerol without CMFs. The physical, chemical, structural, and mechanical properties of the films were evaluated. The optimized formulation (FO), containing 1.00% CPMC and 1.00% CSMC, exhibited a four-fold increase in tensile strength (2.71 MPa) compared to the control. Water vapor permeability was significantly reduced (from 6.33 × 10^−4^ to 2.82 × 10^−4^ gH_2_O·mm/m^2^·h·mmHg), and solubility decreased to 75.82%. Biodegradability was modulated, with FO exhibiting 73.06% degradation over three days versus complete degradation of FC within one day. The incorporation of CMFs, particularly from agro-industrial residues, significantly improved the structural integrity and barrier properties of the films, highlighting their potential for use in biodegradable packaging systems.

## 1. Introduction

Food waste remains a critical global problem, with substantial environmental and economic consequences. In 2022, approximately 1.05 billion tons of food waste were generated worldwide, equivalent to about 132 kg per capita per year [1]. Meanwhile, global polymer production between 2020 and 2024 reached approximately 531 million tons (Mt), of which only 8% (40.8 Mt) came from biobased sources. In the functional and structural polymer segment (410.8 Mt), the biobased content represents only 4% [2].

In response to recycling challenges and growing environmental pollution, interest in biodegradable materials derived from renewable biopolymers, especially those obtained from agro-industrial waste, has grown. In addition to mitigating environmental impacts, these materials promote the value of food byproducts, integrating technological innovation and sustainability [3,4].

The increasing demand for sustainable alternatives to synthetic polymers has catalyzed the development of biodegradable packaging materials based on biopolymers. Among these, cellulose-derived biopolymers, such as microcellulose, nanocellulose, methylcellulose, and carboxymethylcellulose (CMC), stand out as promising candidates. These materials offer a unique combination of renewability, excellent mechanical properties, and environmental degradation, making them particularly attractive for applications in eco-friendly packaging solutions. Especially, microcellulose has emerged as a promising reinforcement in natural matrices such as gelatin [5,6]. Studies show that the incorporation of cellulose microfibrils (CMFs) increases the tensile strength, structural integrity, and moisture barrier of films, making them competitive with synthetic materials [7,8].

The main motivation for replacing petrochemical packaging with renewable sources, like lignocellulosic materials, is the urgent need to reduce the accumulation of plastic waste in the environment and to valorize abundant lignocellulosic materials. However, the compatibility between microcellulose and natural polymers still presents challenges, such as hydrophobicity, heterogeneous dispersion, and limited physical interactions, which compromise the processability and final performance of the films. Therefore, although promising, the industrial application of microcellulose requires surface modification strategies, formulation optimization, and economic viability [9,10].

In this context, films formulated with hemicellulose and methylcellulose or CMC, when combined with appropriate plasticizers, achieve good performance in mechanical, barrier, and optical properties [11]. The combination of microcellulose extracted from lignocellulosic waste with strategies such as surface modification has proven effective in overcoming these challenges, promoting materials aligned with the best use of resources [12,13].

Cellulose, the most abundant natural polymer on Earth, is present in plants, algae, tunicates, and bacteria. It is a homopolysaccharide with the formula (C_6_H_10_O_5_)_n_, composed of β-D-glucopyranose units linked by β-1,4-glycosidic bonds [14,15]. Among the plant residues used to obtain micro- and nanocellulose, corn stover (*Zea mays*) stands out, with approximately 35–40% cellulose [16], and cowpea pod husks (*Vigna unguiculata*), with an approximate 29.97% cellulose content [17].

Due to its renewable and biodegradable nature, cellulose has applications in sectors such as energy, construction, and the textile industry. More recently, it has been used in the development of biodegradable films and composite materials [18]. These films, made from polysaccharides, proteins, or lipids, act as barriers against oxygen, light, and moisture, helping to extend the shelf-life of foods and reduce environmental impacts [19,20].

Gelatin, a protein obtained by the partial hydrolysis of collagen, is widely used in food packaging due to its film-forming properties, biodegradability, and biocompatibility [21]. However, its high water solubility and low thermal stability limit its use. Research has shown that the addition of cellulosic reinforcements can overcome these limitations [22,23]. The incorporation of cellulose into gelatin films promotes the formation of hydrogen bonds, increasing mechanical strength and thermal stability and decreasing solubility, transparency, and water vapor permeability [3,24,25,26,27,28].

Despite recent advances in gelatin film studies, no reports of formulations using cellulose microfibrils extracted from cowpea pod hulls have been found. Nor have formulations combining CMFs from this source (CPMC) with those obtained from corn stover (CSMC) in the same film. Furthermore, optimization studies of these formulations are still limited. This research hypothesized that the production of gelatin films containing CPMC and CSMC could improve their structural, thermal, and morphological properties. Therefore, the objective of the study was to develop and characterize gelatin films reinforced with CPMC and CSMC, evaluating their physical, chemical, mechanical, and thermal properties.

## 2. Materials and Methods

### 2.1. Preparation of Microcelluloses

Cowpea pods (*Vigna unguiculata*) and corn straw (*Zea mays*) were obtained at street markets in João Pessoa, Paraíba, Brazil, during the period of February to July 2024. The plant materials were selected through a visual analysis of their physical integrity and then washed with detergent and running water. The skins and straws were spread in a thin layer on a metal tray and subjected to oven drying at 50 °C with forced air circulation until weight stabilization [29]. Finally, the dried skins and straws were ground in a knife mill, sealed under vacuum, and refrigerated at 4 °C until the subsequent steps.

The process of obtaining CMFs from cowpea pod skins (*Vigna unguiculata*) and corn straw (*Zea mays*) followed the methodology described by Souza et al. [8]. Initially, 20 g of each biomass was subjected to delignification using 200 mL of a 50/50% (*v*/*v*) ethanol/distilled water mixture. Next, 200 mL of 5% (*v*/*v*) aqueous sulfuric acid was added under stirring to complete the delignification process. The biomasses were washed with ethanol and distilled water until neutralization. After the removal of hemicellulose and lignin, the biomasses were bleached by stirring with a 24% (*v*/*v*) hydrogen peroxide solution and a 4% (*m*/*v*) sodium hydroxide solution, both mixed in a 1:10 (g/mL) ratio. Finally, microcellulose was obtained through ball milling for 15 h, assisted by acid hydrolysis with a 30% (*v*/*v*) aqueous sulfuric acid solution.

### 2.2. Experimental Design and Film Preparation

Preparation of the films was carried out using the molding method reported by Freire et al. [30]. Initially, the CMFs extracted from corn straw and cowpea pod skins were diluted in an alcoholic solution (1 g/10 mL). Then, gelatin (2%, *w*/*w*), glycerol (2%, *w*/*w*), and the alcoholic solution containing the CMFs at concentrations of 0.00, 0.29, 1.00, 1.71, and 2.00% (*w*/*w*) were added to water. Homogenization of the mixture was carried out for 40 min at 60–70 °C with constant stirring at 600 rpm on a magnetic stirrer. The mixture was dispensed into acrylic Petri dishes (150 mm × 15 mm) for film formation with a constant weight of 45 g. The samples were dried in a circulating oven at 40 °C for 20–22 h, then conditioned in silica gel desiccators before testing.

The film formulations were developed using a 2^2^ experimental design. In total, 11 experiments were carried out, incorporating 4 axial points and 3 central replicates. The independent variables were CSMC and CPMC. The range of these variables was established based on preliminary studies, with concentrations set at 0.00, 0.29, 1.00, 1.71, and 2.00% (*w*/*w*). The response variables included tensile strength (TS), elongation at break (EB), and water vapor permeability (WVP). The response surface was generated using an empirical complete quadratic polynomial model, as represented by Equation (1), where β denotes the model coefficients. The design and data analysis were performed using Statistical Software (version 7).

*Y* = β0 + β_1_
*X*_1_ + β_2_
*X*_2_ + β_12_
*X*_1_
*X*_2_
*+* β_11_
*X*_1_^2^ ± β_22_
*X*_2_^2^ + *e* (1)

The response variables TS, EB, and WVP were measured under optimized conditions for model validation. Once validated, the optimized film was thoroughly characterized, including physico, chemical, mechanical, morphological, and thermal analysis.

### 2.3. Film Characterization

#### 2.3.1. Thickness and Mechanical Properties: Tensile Strength (TS) and Elongation at Break (EB)

Film thickness was measured at room temperature (25 °C). A portable micrometer (accuracy: 0.001 mm) was used to take ten random measurements at a distance of 60 mm from the edges of the films. Subsequently, the average of the ten measurements was determined in millimeters.

The tensile strength of the film and the elongation at break were evaluated using a static testing instrument (SHIMADZU, Kyoto, Japan), following American Society for Testing and Materials (ASTM) standard ASTM D882-12 [31]. The samples were cut into rectangular shapes measuring 100 mm × 15 mm, employing an initial grip distance of 50 mm and a testing rate of 12.5 mm/min. Ten measurements were performed for each formulation. Results were presented as megapascals (MPa) for tensile strength and percentage (%) for elongation at break.

#### 2.3.2. Water Vapor Permeability (WVP)

The films’ water vapor permeability was assessed using the ASTM E96-95 method [32]. The 5 cm diameter films were placed in polyethylene containers, sealed with silica gel inside. These containers were kept in a desiccator humidified with saturated NaCl solution, maintaining a temperature of 25 °C and 75% relative humidity. The weights of the samples were determined every 24 h for one week using an analytical balance, with four replicates for each measurement. The data were expressed in (gH_2_O mm/m^2^·h·mmHg) and calculated using Equation (2):

(2) $$WVP\;=\;\left[\left(C_{i}/A\right)X\right]/\left[P_{s}\left({RH}_{1}\;-\;{RH}_{2}\right)\right]$$

where C_i_ (slope of the line obtained from the system’s weight gain over time); A (film’s area, m^2^); X (film thickness, mm); P_s_ (stands for the saturation pressure of water vapor at 25 °C, mmHg); RH_1_ (relative humidity inside the chamber, 75%); and RH_2_ (relative humidity within the capsule, 0%).

#### 2.3.3. Color and Opacity

Film color was measured using a Konica Minolta CR400 colorimeter (Konica Minolta, Osaka, Japan). The L* (lightness), a* (red-green), and b* (yellow-blue) parameters were directly recorded according to the CIE Lab system. The colorimeter was calibrated using a standard white calibration plate. The total color difference (ΔE) was calculated using Equation (3), with FC serving as the reference, following Lee et al. [33]:

(3) $$\Delta E={\left[\left({\left(L^{*}-L\right)}^{2}+{\left(a^{*}-a\right)}^{2}\;+\;{\left(b^{*}-b\right)}^{2}\right)\right]}^{1/2}$$

Film opacity was measured following the procedure outlined by Lee et al. [33]. The films were cut into strips measuring 25 mm × 1 mm and inserted into quartz cuvettes, which were then positioned perpendicular to the incident light beam for opacity analysis. An empty quartz cuvette was used as a blank reference during all measurements. Opacity was measured using a spectrophotometer (UV-2450, Shimadzu Corporation, Kyoto, Japan) and calculated according to Equation (4), where Abs_600_ is the absorbance at 600 nm and x represents the film thickness:

(4) Opacity=Abs600/x

#### 2.3.4. Solubility

Film samples, cut into 2 cm × 2 cm squares, were dried in a circulating oven at 105 °C for 24 h. Subsequently, the samples were submerged in 50 mL of water inside a 250 mL Erlenmeyer flask and stirred at 100 rpm using a MultiShaker for 24 h at 25 °C. Afterward, the water was removed by drying the samples in an air circulation oven at 105 °C for 24 h, and the dry matter mass was measured. Solubility was expressed as a percentage (%) and calculated using Equation (5) [34], where P_is_ is the initial mass of the dry material and P_fs_ is the final mass of the dry material that did not dissolve:

(5) $$Solubility\;\left(\%\right)=\left[\left(P_{is}-P_{fs}\right)/P_{is}\right]\;\times \;100$$

#### 2.3.5. Fourier Transform Infrared Spectroscopy (FTIR)

The analysis to determine functional groups by FTIR was carried out using a Shimadzu IR Prestige-2 spectrophotometer (Shimadzu, Kyoto, Japan) with an attenuated total reflectance (ATR) accessory. The films were positioned directly on the sample holder at 25 °C, and spectra were collected in transmittance mode across the 600–4000 cm^−1^ range, with a resolution of 4 cm^−1^ and 40 scans [35]. All spectra underwent auto-baseline correction before curve fitting. The FTIR spectra was generated using IR Solutions software (version 2), and peak fitting was performed using OriginPro software (version 8.5; OriginLab Corporation, Northampton, MA, USA).

#### 2.3.6. Degradability

The degradation of the films was evaluated using a method adapted from Carli et al. [36] that involved measuring mass loss as samples were exposed to soil and its indigenous microbiota over 1-, 2-, and 3-day intervals. Samples were cut into 2 cm × 2 cm rectangles, weighed, and buried at a depth of 15 cm in polyethylene containers. A total of 10 mL of water was added to the samples at 24 h. At the same interval, films were excavated, rinsed with distilled water to remove debris, dried in a circulating oven at 40 °C for 24 h, and then reweighed. The percentage of degradation was calculated using Equation (6), where mi represents the initial mass of the film (g) and mf represents the final dry mass (g):

(6) Degradability (%) = [(mi − mf)/mi] × 100

#### 2.3.7. Thermogravimetric Analysis (TGA/DTG)

The films were analyzed by thermogravimetric analysis (TGA) using a Shimadzu DTG-60H thermal analyzer. Approximately 5–10 mg of the sample was placed in an alumina crucible and the analysis was conducted under a nitrogen atmosphere with a flow rate of 50 mL/min. The heating rate was set to 10 °C/min, covering a temperature range from 30 °C to 900 °C. The resulting thermograms, which displayed the percentage of weight loss as a function of temperature, were analyzed using OriginPro 8.5 from OriginLab Corporation, Northampton, MA, USA.

#### 2.3.8. X-Ray Diffraction (XRD)

The XRD analysis was performed following the methodology outlined by Spagnol et al. [37]. The XRD analysis was performed using a Shimadzu XRD-6000 diffractometer (Shimadzu, Tokyo, Japan), with an applied voltage of 30 kV and a current of 30 mA (Cu Kα radiation, λ = 1.5418 Å). The scanning range covered 2θ angles from 10° to 50°, with a scanning speed of 2°/min. OriginPro software (version 8.5, OriginLab Corporation, Northampton, MA, USA) was used to analyze the resulting diffractograms.

#### 2.3.9. Scanning Electron Microscopy (SEM)

Samples were examined using a MIRA-3 LMH microscope (Tescan, São Paulo, SP, Brazil) featuring a high-brightness font and functioning under high vacuum. The films were secured onto aluminum holders with double-sided carbon tape and coated with a gold layer to ensure conductivity. Imaging was conducted using MiraTC software (version 3) at an accelerating voltage of 5 kV, employing secondary electron (SE) detectors set to a flux of 1750 mA, and magnifications ranging from 500× to 10,000×.

#### 2.3.10. Statistical Analysis

Values are expressed as the mean with the corresponding standard deviation, presented in tables, graphs, and images. Experiments were performed in triplicate to ensure accuracy, except for the mechanical properties and water vapor permeability tests. The experimental design data were analyzed through regression coefficients to create response surfaces using Statistical Software (version 7). The impacts of the independent variables were assessed, and the desirability test was applied to determine the optimal film production conditions. Validation of the mathematical model was carried out by replicating formulations under the optimal conditions proposed by the desirability function [38].

## 3. Results and Discussion

### 3.1. Film Production Conditions

Table 1 presents the experimental design matrix used to evaluate the effects of two independent variables: X_1_ (corn straw microcellulose, CSMC) and X_2_ (cowpea pod skin microcellulose, CPMC). These variables were assessed in relation to three response variables: TS (tensile strength), EB (elongation at break), and WVP (water vapor permeability).

Table 1 demonstrates that changes in CSMC and CPMC concentrations had a significant impact on the mechanical and barrier properties of the gelatin-based films. Specifically, formulations with lower microcellulose content exhibited a marked decrease in tensile strength (TS), such as formulations 1 (0.29% CSMC; 0.29% CPMC), 5 (0.00% CSMC; 1.00% CPMC), and 7 (1.00% CSMC; 0.00% CPMC). By contrast, the highest TS value was observed in formulation 6 (2.00% CSMC; 1.00% CPMC). This enhancement was attributed to the increased microcellulose content, which reinforced the polymeric matrix by improving the interfacial interactions between CMFs and the gelatin–glycerol system—likely through covalent bonding. In addition to these interfacial bonds, factors such as the degree of CMF dispersion also contributed to the observed increase in TS [39]. Conversely, the highest elongation at break (EB) was found in formulation 1, which contained the lowest levels of CSMC and CPMC. This suggested that lower concentrations of microcellulose reduced the density of strong interfacial bonds, enhancing polymer chain mobility and thereby increasing flexibility and elongation capacity [40].

The lowest water vapor permeability (WVP) values were recorded in formulations 9, 10, and 11—central points of the experimental design—containing 1.00% of both CSMC and CPMC, with values ranging from 2.80 to 2.83 × 10^−4^ gH_2_O·mm/(m^2^·h·mmHg). Although formulations 3 (1.71% CSMC; 0.29% CPMC) and 6 (2.00% CSMC; 1.00% CPMC) exhibited high TS values (3.10 and 4.42 MPa, respectively), they also showed increased WVP and reduced EB.

Notably, formulation 7 exhibited the lowest WVP value among all samples, despite containing only one source of CMFs. The central formulations (9–11) also demonstrated low WVP values, possibly due to synergistic effects between CSMC and CPMC at intermediate concentrations. Factors influencing WVP include the hydrophilic/hydrophobic balance of the film matrix, film thickness, surface roughness, material crystallinity, and the size, dispersion, and orientation of CMFs. Additionally, greater chain mobility and weaker interactions between CMFs and gelatin can contribute to increased WVP [41,42].

**Figure 1:**
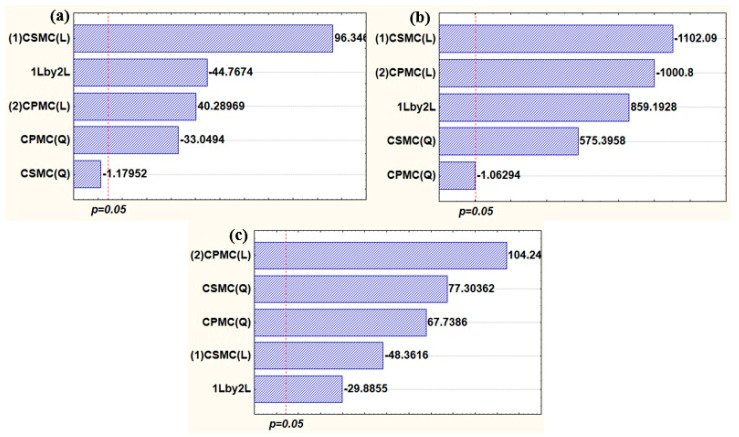
Pareto charts of the response variables: (**a**) tensile strength—TS; (**b**) elongation at break—EB; and (**c**) water vapor permeability—WVP. Note: Independent variables CSMC (corn straw microcellulose content); CPMC (cowpea pod skin microcellulose content). Pareto chart shows the effects of the formulation variables on the measured response. CSMC (L) and CPMC (L) indicate linear effects, and CSMC (Q) and CPMC (Q) indicate quadratic effects of corn straw microcellulose content and cowpea pod skin microcellulose content, respectively. 1Lby2L represents their linear interaction. Effects beyond the red line are statistically significant (*p* < 0.05).

Figure 1 shows the analysis of the Pareto charts, constructed based on the experimental data obtained during the optimization process, which reveal the effects of the factors on the responses of each response variable: tensile strength (TS), elongation at break (EB), and water vapor permeability (WVP).

The Pareto chart in Figure 1, generated from the experimental design data during the optimization process, highlights the significant effects of the independent variables on each response variable. As shown in Figure 1a, the tensile strength (TS) response was linearly influenced by both CSMC and CPMC, with CSMC exerting a stronger and positive effect. By contrast, the quadratic term of CPMC demonstrated a negative effect, indicating that higher concentrations of CPMC led to a reduction in the TS of the films. Furthermore, the interaction between the two types of CMFs exhibited a negative synergistic effect on this response. Based on these results, it could be inferred that the addition of both CMFs played a positive role as reinforcing agents in gelatin-based films due to their effective interaction with the film matrix [39].

Figure 1b shows that elongation at break (EB) was negatively affected by both CSMC and CPMC, suggesting that higher concentrations of CMFs resulted in reduced film flexibility. However, regarding the quadratic effect, increasing the concentration of CSMC had a positive impact on EB. Additionally, a synergistic interaction between CSMC and CPMC resulted in a positive effect on this response. The reduction in EB was expected with the incorporation of CMFs, as the bonds formed between the reinforcing material and the gelatin matrix chains reduced the film’s flexibility and elongation capacity [40].

Figure 1c shows that for the water vapor permeability (WVP) response function, CSMC had a positive linear effect, while CPMC exhibited a negative linear effect. Regarding the quadratic effects, both CSMC and CPMC contributed positively to WVP. Lastly, the synergistic interaction between CSMC and CPMC had a negative impact on the WVP response function. The reduction in WVP due to the synergistic interaction between the CMFs in the film formulation was chemically expected. This was because improved hydrogen bonding interactions between the CMFs and the gelatin matrix reduced chain mobility, thereby decreasing WVP [41,42].

**Figure 2:**
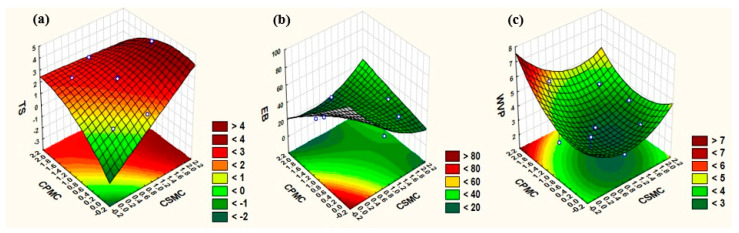
Response surface generated for the response variable: tensile strength—TS (**a**); elongation at break—EB (**b**); and water vapor permeability—WVP (**c**). Note: CSMC (corn straw microcellulose content); CPMC (cowpea pod skin microcellulose content); TS (tensile strength); EB (elongation at break); WVP (water vapor permeability).

Figure 2 presents the response surfaces for the variables tensile strength—TS, elongation at break—EB, and water vapor permeability—WVP. These surfaces were generated to evaluate the significance of using CMFs in films with respect to these parameters. The response surface plots generated for the response variables provide a simplified visualization of how the concentrations of CPMC and CSMC affect each parameter. In Figure 2a, tensile strength (TS) increases with higher concentrations of both CPMC and CSMC, confirming their reinforcing role in the material. In Figure 2b, elongation at break (EB) decreases as the concentrations of CPMC and CSMC increase, consistent with the expected reduction in flexibility. Finally, Figure 2c shows that water vapor permeability (WVP) increases with increasing CPMC concentration.

The response surface analysis for each of the evaluated variables resulted in Equations (7)–(9), which represent the predicted values for tensile strength (TS), elongation at break (EB), and water vapor permeability (WVP), respectively. Based on these equations and the response surface plots, the concentrations of X_1_ (CPMC) and X_2_ (CSMC) had a significant influence on the analyzed parameters. The analysis indicated that the optimal film performance was achieved at intermediate concentrations of both CPMC and CSMC.

(7) $$TS=-0.86+2.38X_{1}-0.02X_{1}^{2}+3.03X_{2}-0.70X_{2}^{2}-1.12X_{1}X_{2}+0.0$$

(8) $$EB=78.55-47.53X_{1}+9.67X_{1}^{2}-27.10X_{2}-0.02X_{2}^{2}+17.12X_{1}X_{2}+0.0$$

(9) $$WVP=3.80-1.90X_{1}+0.99X_{1}^{2}-0.49X_{2}+0.87X_{2}^{2}-0.45X_{1}X_{2}+0.0$$

where X_1_ (corn straw microcellulose, CSMC); X_2,_ (cowpea pod skin microcellulose, CPMC) content; TS (tensile strength); EB (elongation at break); WVP (water vapor permeability)

**Figure 3:**
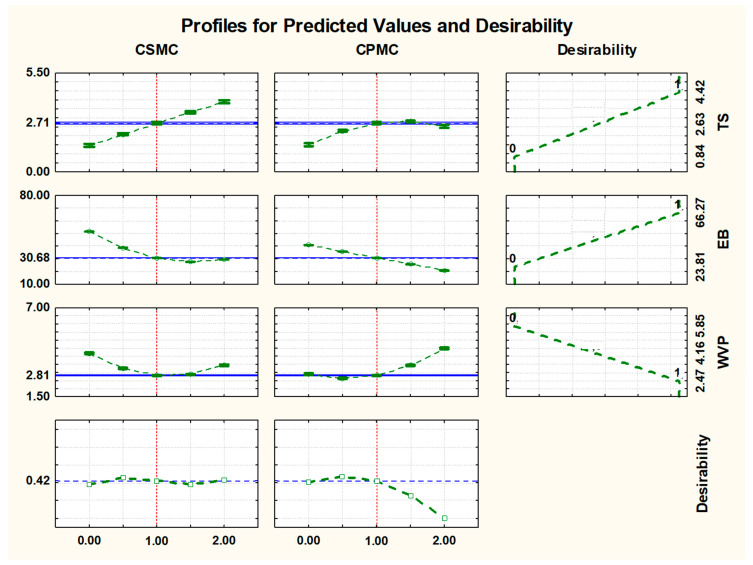
Predicted value and desirability profile. Note: CSMC (Corn straw microcellulose); CPMC (Cowpea pod skin microcellulose); TS (tensile strength); EB (elongation at break); WVP (water vapor permeability). Effect of CSMC and CPMC levels on the global desirability function, combining individual desirabilities of TS, EB, and WVP (0 = completely undesirable; 1 = fully desirable). The third plot in the last row shows the overall desirability.

The application of the desirability function in Figure 3 revealed that the region containing trials 9, 10, and 11 (central points) was within the range of 1.0% CSMC and 1.0% CPMC. These values aligned with the optimized variables predicted by the response surface analysis. The central point demonstrated superior quality attributes and was deemed the formulation that best fulfilled the optimal conditions for film fabrication. Then, the film was validated based on the conditions identified for desirability.

Figure 3 shows the predicted value and desirability profile for the following parameters: tensile strength (TS), elongation at break (EB), and water vapor permeability (WVP).

For the TS parameter, when using the response surface for variables X_1_ = 1.00% CSMC and X_2_ = 1.00% CPMC, the measured TS value (2.71 MPa) was compared with the predicted TS value (2.71 MPa) and no significant difference was observed between the means. Similarly, for EB, the mean of the repetitions (30.68%) and the predicted value (30.69%) were nearly identical. For WVP, no meaningful difference was detected between the average of the repetitions (2.82 × 10^−4^ gH_2_Omm/m^2^h.mmHg) and the predicted value (2.81 × 10^−4^ gH_2_O.mm/m^2^h.mmHg). Given these results, the film with 1.00% CSMC and 1.00% CPMC was selected for further characterization.

### 3.2. Characterization of the Films

The parameters evaluated for both samples (optimized and control formulations) allowed for a comparison of the changes in film properties resulting from the incorporation of CMFs, compared to those of films without CMFs (Table 2). The results obtained indicated significant differences in the properties of the biodegradable films with the addiction of CMFs, with (1.00% CSMC and 1.00% CPMC) and without the addition of CMFs, highlighting the positive impact of the bioactive extract on the polymer matrix.

Compared to the control formulation (FC), the optimized formulation (FO) exhibited a tensile strength approximately four times higher. A similar enhancement in TS was reported in sodium alginate films reinforced with nanocrystalline cellulose extracted from grape residues [43]. Likewise, chitosan-based films incorporating nanocellulose derived from soybean straw exhibited a 2.3-fold increase in TS compared to control films without cellulose [44]. By contrast, films containing 2–5% cellulose nanofibers (CNF) from commercial sources showed a more moderate increase in TS, ranging from 40–60% relative to unreinforced films [25].

Even with the observed enhancements, the TS values were lower than those typically reported for conventional plastics, such as low-density polyethylene (LDPE) and polyvinyl chloride (PVC), which typically exhibit TS values ranging from 8.34 to 36.1 MPa [45]. Nonetheless, the incorporation of lignocellulosic materials clearly enhanced the TS of gelatin films. This improvement was likely due to covalent bonding between the gelatin matrix and cellulose microfibers (CMFs). Additionally, CMFs can act as reinforcing microfibers and potential crosslinking agents, contributing to stronger hydrogen bonding and hydrophobic interactions, which further enhance mechanical properties [46].

The elongation at break (EB) of FO was lower than that of FC, reflecting the stiffening effect imparted by the CMFs. This reduction in film ductility could be explained by the decreased cohesion between gelatin polymer chains as a consequence of the constrained interaction between the matrix and the reinforcing fibers [47]. The EB values obtained for FO were significantly lower than those reported for LDPE (627%) and PVC (440.9%) [45]. A similar decline in EB following the incorporation of lignocellulosic materials was documented in poly(lactic acid)/gelatin films reinforced with nanocrystalline cellulose (CNC) and antioxidant extracts from almond shells [26], as well as in CNF-reinforced films using commercial cellulose [13] and sodium alginate films reinforced with grape-derived nanocellulose [43].

Regarding water vapor permeability (WVP), FO exhibited lower values than FC, although both remained higher than those typical of commercial plastics. For example, LDPE films typically display a WVP of 0.80 × 10^−4^ g·mm/m^2^·h·mmHg [48]. The reduction in WVP in the reinforced films was probably a consequence of the hydrophobic characteristics of CMFs. This trend aligned with previous findings in CNC-reinforced films, where WVP was reduced from 17.42 to 8.64 × 10^−3^ g·mm/m^2^·h·mmHg [27].

In gelatin-based films, the improved barrier properties can be associated with better dispersion of microfibers within the polymer matrix, creating tortuous paths that hinder water vapor transmission. Additionally, the reduced mobility of protein chains and strong interactions between CMFs and the gelatin–glycerol network contribute to decreased water diffusivity. These interactions promote hydrophobicity, thereby enhancing the film’s resistance to moisture transfer [49].

The thickness of the optimized formulation (FO) was greater than that of the control formulation (FC), indicating that the incorporation of cellulose microfibers (CMFs) affected this parameter—likely due to the limited solubility and dispersion of CMFs in the film-forming solution. Onyeaka et al. [25] reported that gelatin–glycerol films had a baseline thickness of 0.15 mm, which increased to 0.16–0.17 mm with the addition of 2–5% nanocellulose. Similarly, in fish gelatin films incorporating Satureja khuzestanica essential oil and bacterial nanocellulose, thickness ranged from 0.14 to 0.16 mm, compared to 0.11 mm for films without bacterial nanocellulose [50].

Packaging film thickness varies according to the polymer type and end-use, with values for conventional polymers such as Polyvinyl chloride (PVC), polyethylene (PE), and polypropylene (PP) typically ranging from 0.050 to 0.10 mm [51]. The thickness of FC aligned with this range, while FO exceeded it. The observed increase in thickness for FO may be attributed to the higher solid content introduced by CMFs, increased viscosity of the film-forming solution, and possible fiber aggregation. These factors contributed to non-uniform dispersion and film heterogeneity [25,52].

In terms of optical properties, the opacity of FO was approximately five times higher than that of FC. Dakhili et al. [50] observed a similar trend, with opacity rising from 0.12% in the control to 0.55% in films containing 4% bacterial nanocellulose. This increase in opacity was largely due to enhanced light scattering caused by higher concentrations of CMFs. Tessaro et al. [44] also reported an increase in opacity from 2.20% to 4.80% upon the addition of 4.5% nanocrystalline cellulose (CNC) extracted from soybean straw.

The increased opacity of gelatin-based films with CMFs may be attributed to the cellulose content, increased crystallinity, aggregate formation, and structural heterogeneity, all of which reduce light transmittance [53]. Opacity values for conventional packaging materials—both bio-based (e.g., cellulose acetate, calcium alginate) and petroleum-based (e.g., polystyrene, PP, LDPE)—typically range from 1 to 5 [54]. The opacity observed for FO fell within this range, indicating that it may be a viable alternative for sustainable packaging applications.

Both the FO and FC formulations exhibited high luminosity (L*) values above 80%, indicating overall brightness. However, the FO sample appeared darker than the FC sample, as reflected by its lower L* value. This reduction in luminosity may be attributed to residual pigments present in the CMFs, which absorb light across various wavelengths and reduce overall reflectance [55].

Comparable results were observed in gelatin-based films reinforced with CNC and antioxidant extract from almond shell, with an L* value of 83.94, and in gelatin–chitosan–glycerol films reinforced with 4.5% CNC from soybean straw, which exhibited an L* value of 87.10 [44]. These findings further support the impact of lignocellulosic fillers on the optical properties of biopolymer films.

The films’ visual characteristics were strongly influenced by the yellow–blue chromatic component (b*). The FO sample exhibited a more intense yellow hue compared to the FC sample, which displayed a lighter yellow coloration. By contrast, the red–green component (a*) had a negligible impact on film coloration. The FO sample demonstrated a slight shift toward the red spectrum, whereas the FC sample tended slightly toward green. This indicated that while the FC film possessed a faint greenish tint, the FO sample remained closer to a neutral tone in the red–green axis.

The ΔE value representing the total color difference between the two samples was 11.61, signifying a clearly perceptible and visually significant alteration in color for the FO film relative to the FC film. According to Romano et al. [56], ΔE values between 2 and 10 are generally visible to the human eye, with values above 10 indicating a strong and noticeable color change. Thus, the FO films demonstrated a marked visual distinction primarily influenced by the L* (lightness) and b* (yellow–blue) components.

Comparable ΔE values were reported in films composed of sodium alginate, gelatin, and CNC derived from grape juice residues, with differences ranging from 2.50 to 29.60 depending on CNC concentration [43]. Similarly, in films incorporating gelatin, chitosan, glycerol, and 4.5% CNC extracted from soybean straw, a ΔE of approximately 8.6 was observed [44], indicating a comparable degree of visual change to that observed in this study.

Both the FC and FO films displayed high water solubility; the control film (FC) exhibited approximately 90% solubility. However, the incorporation of CMFs into the FO formulation led to a solubility reduction of about 14%. This decline may have resulted from the presence of water-insoluble substances such as cellulose and its derivatives, which reduced the film’s overall affinity for water. Similar trends were reported in gelatin films reinforced with 4.5% CNC and almond shell extract, which showed a 7% reduction in solubility compared to films without CNC [25]. In gelatin/agar-based films containing commercial CNFs and clove essential oil, solubility values were even lower, ranging from 3% to 10% [57].

The observed reduction in solubility upon CMF incorporation could be attributed to enhanced hydrogen bonding between the gelatin polymer chains and the lignocellulosic fibers. These interactions contributed to a more cohesive and less water-permeable matrix, strengthening the structural integrity of the film. Moreover, the hydrophobic nature of CMFs increased the water resistance of the composite material by limiting the infiltration and dissolution of water molecules within the polymer network [58,59].

### 3.3. FTIR Spectrophotometry of the Films

**Figure 4:**
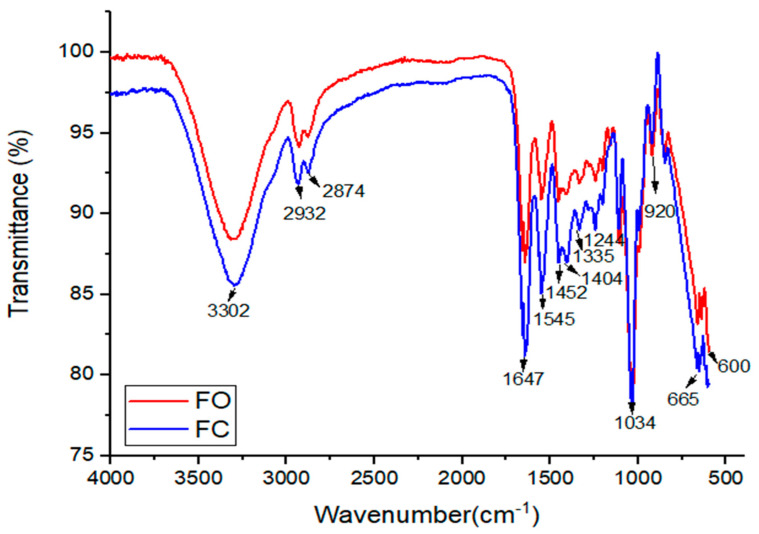
FTIR spectra of films with CMFs (FO) and without CMFs—control (FC). Note: FO (film optimal); FC (film control).

The Fourier transform infrared (FTIR) spectra of the films with CMFs (FO) and the control film without CMFs (FC) are shown in Figure 4. A comparison of the spectra reveals that the incorporation of CMFs led to slight shifts in several characteristic absorption bands. Specifically, the peaks observed at 2932, 2874, 1647, 1545, and 1244 cm^−1^ in the FC film shifted to 2920, 2870, 1632, 1543, and 1234 cm^−1^, respectively, in the FO film. While the majority of the bands remained similar between the two samples, these subtle spectral changes suggested chemical modifications resulting from the integration of CMFs into the polymer matrix. These modifications likely reflected the formation of intermolecular interactions, such as hydrogen bonding, between the CMFs and the gelatin–glycerol matrix [60].

A strong and broad absorption band centered around 3302 cm^−1^ was observed in both FC and FO films. This absorption band generally arises from O–H stretching vibrations, reflecting hydroxyl groups present in cellulose, gelatin, and glycerol molecules [61]. Additionally, two adjacent peaks at approximately 2932 and 2874 cm^−1^ (FC) and 2920 and 2870 cm^−1^ (FO) were detected, which are characteristic of the asymmetric and symmetric stretching vibrations of –CH groups in saturated carbon chains. These bands further confirmed the presence of cellulose-based and proteinaceous materials within the film structure.

The observed spectral shifts and consistent band features supported the hypothesis that CMFs were successfully incorporated into the film matrix, promoting molecular interactions without disrupting the overall polymer structure. These interactions contributed to the modified physicochemical and mechanical properties observed in the FO films.

In the FTIR spectra, the peak observed at 1034 cm^−1^ corresponded to C–O stretching vibrations, which are characteristic of alcohol and ether functional groups. A distinct peak near 1100 cm^−1^ corresponded to C–O–C stretching vibrations, indicative of glycosidic linkages commonly found in polysaccharide structures derived from cellulose. The presence of these bands confirmed the contribution of cellulose-based materials to the film matrix [50].

A prominent absorption band around 1647 cm^−1^ corresponded to C=O stretching vibrations, associated with the carboxylate group (COO^−^) within the amide I region of the gelatin structure. This strong bond is known to enhance intermolecular interactions, potentially contributing to improved tensile strength in the films. Furthermore, the peak observed at 1545 cm^−1^ corresponded to the overlapping stretching and bending vibrations of N–H and C–N bonds, indicative of the amide II region. These signals are associated with primary and secondary amides in the gelatin matrix [50].

These spectral features were consistent with previous findings in films composed of gelatin, chitosan, glycerol, and CNC extracted from soybean straw, where the incorporation of nanocellulose at concentrations below 5% by weight resulted in minimal changes to the FTIR spectra [44]. This suggested that while CMFs contributed to the mechanical reinforcement of the films, their presence did not significantly alter the overall chemical structure, as detected by FTIR at low concentrations.

### 3.4. Soil Degradation

Biodegradation of polymeric materials typically proceeds through three sequential phases, as outlined by Baranwal et al. [62]: (i) colonization of the plastic surface by native soil microorganisms leading to biofilm formation; (ii) secretion of extracellular enzymes that catalyze the depolymerization of the polymer matrix into lower molecular weight oligomers and monomers; and (iii) assimilation of these degradation products by the microorganisms as a nutrient source.

In the present study, the control film (FC) exhibited complete biodegradation within 1 day of soil burial, whereas the film containing cellulose microfibers (FO) demonstrated a 73.06% reduction in mass after 3 days. This slower degradation of FO can be attributed to the structural complexity and physicochemical properties introduced by the addition of lignocellulosic material. The literature reports analogous results, including gelatin-based films reinforced with cellulose nanofibers (CNF) derived from rice straw (2.5% and 5.0%) showed weight reductions of approximately 35–36% after 3 days, 71–75% after 5 days, and up to 93–97% after 7 days [24]. Similarly, gelatin films reinforced with CNFs extracted from post-consumer wet wipes exhibited weight losses of 61.18%, 71.80%, and 79.90% after 7 days for films containing 5%, 10%, and 15% CNC, respectively [3].

On the other hand, typical plastic films such as polyethylene (PE), polypropylene (PP), and polyvinyl chloride (PVC) degrade at a substantially slower rate, with mass losses typically ranging from only 10% to 28.4% over a 90-day period under similar soil burial conditions [63]. This highlights the potential environmental advantage of biodegradable biopolymer films.

The slower degradation of the FO film, compared to the FC film, was primarily linked to the intrinsic properties of cellulose. Cellulose possesses an amphiphilic nature due to the presence of both hydrophilic hydroxyl (–OH) and hydrophobic methylene (–CH) groups. Its crystalline regions, stabilized by strong C–C and C–O covalent bonds and intra/intermolecular hydrogen bonding, reduce its accessibility to microbial enzymes [64]. The hydrophobic interactions between glucopyranose units further promote the formation of tightly packed, sheet-like structures, which are more resistant to enzymatic attack. As a result, the incorporation of cellulose microfibers enhanced the structural integrity and water resistance of the films, thereby delaying microbial colonization and subsequent degradation. This explained the higher degradation resistance observed in FO films relative to FC films.

### 3.5. Thermal Properties

**Figure 5:**
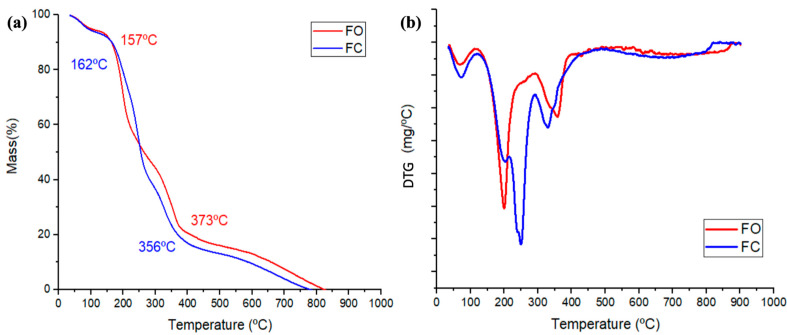
(**a**) TGA curves of the films; (**b**) DTG curves of the film). Note: FO (film optimal); FC (film controls.

Figure 5 presents the thermal analyses of films with CMFs (FO) and without CMFs (FC). Figure 5a displays the TGA curves, while Figure 5b shows the corresponding DTG curves.

Thermogravimetric analysis (TGA) revealed that the thermal degradation of the FO film occurred in three distinct stages, whereas thermal analysis revealed four degradation stages for the control film (FC). The initial degradation event (69–73 °C) was associated with the evaporation of moisture and volatile compounds. FO exhibited a lower mass loss in this stage compared to FC, likely due to the enhanced water retention conferred by the cellulose microfibers (CMFs). The second degradation stage, occurring around 200 °C in both the FO and FC films, was due to the thermal degradation of glycerol, which begins to degrade at approximately 182 °C [65].

In the FC formulation, an additional third stage was observed at 249 °C, corresponding to the evaporation of residual glycerol and the decomposition of low-molecular-weight fractions of gelatin [3,66]. The final degradation phase, observed between 327–357 °C in both FO and FC samples, was associated with the breakdown of the protein matrix and, in the case of FO, the degradation of CMFs.

Interestingly, the FO film displayed an upward shift in the temperature range of this final degradation stage, indicating enhanced thermal stability. This behavior was attributed to the incorporation of CMFs, which likely formed hydrogen bonds with the gelatin matrix. Such interactions may promote the development of triple-helix structures, altering the protein denaturation pathway and thereby delaying thermal degradation [66]. Additionally, the higher thermal stability observed in FO may have resulted from the increased interfacial area between the gelatin matrix and the cellulose network, which hindered thermal decomposition by creating a more cohesive and cross-linked structure [67].

For instance, films composed of gelatin, agar, glycerol, and 12% CNFs extracted from rice straw exhibited a maximum degradation temperature of 246 °C [24], while bovine gelatin-based films reinforced with 2.5% CNC from eucalyptus pulp reached a maximum of 216 °C, compared to 206 °C for the unreinforced control [68]. Similarly, sodium alginate-gelatin films containing 0.5–1% CNC derived from grape juice residue demonstrated maximum degradation temperatures ranging from 180 to 250 °C [43]. The thermal stability values obtained in this study fell within a similar range.

It is important to note that thermal stability may vary due to multiple factors, including the concentration, morphology, and dispersion of cellulose fibers, as well as the interaction dynamics within the polymer matrix. Although the addition of CMFs enhanced the maximum degradation temperature, it also led to a reduction in the onset temperature (Tonset). This suggested a disruption in the original gelatin–glycerol interactions, potentially due to steric hindrance imposed by the fibrous cellulose structure [25].

### 3.6. Structural Characterization by X-Ray Diffraction (XRD)

**Figure 6:**
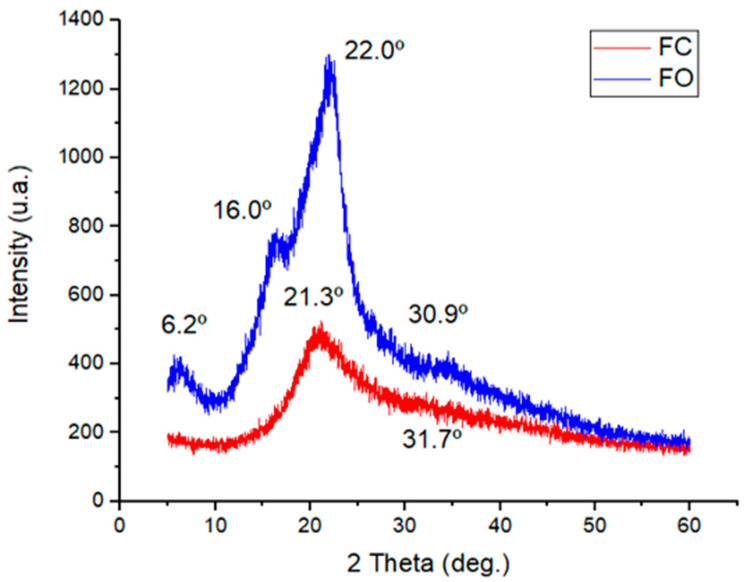
XRD curves of films with CMFs (FO) and without CMFs (FC). Note: FO (film optimal); FC (film control).

As illustrated in Figure 6, the X-ray diffractograms were compared for films with CMFs (FO) to those without CMFs (FC).

The X-ray diffraction (XRD) pattern of the FO formulation exhibited distinct peaks at 6.4°, 16.0°, 22.0°, and 30.9°, characteristic of cellulose polymorph I, confirming the presence of highly CMFs content [69]. The incorporation of CMFs into the gelatin-based films led to noticeable shifts in peak positions and intensities, reflecting significant structural changes within the film matrix. These changes could be attributed to the high degree of crystallinity associated with CMFs, which enhanced the overall crystallinity index and rigidity of the composite films [70].

By contrast, the control formulation (FC), lacking CMFs, exhibited lower peak intensity and less defined crystalline structures. Notably, the peak observed around ~6.2° was associated with the triple-helix conformation typical of gelatin [71]. The area under this peak has been shown to correlate directly with the triple-helix content in gelatin-based composites [72]. In the FO films, this band shifted position slightly, suggesting the disruption or reorganization of the gelatin helix structure as a result of interactions with the cellulosic components. The degree of this shift appears to depend on the concentration and distribution of CMFs within the film matrix [27].

Peaks at 22.0° and 30.9° in the FO sample were consistent with those reported by Nikoukheslat et al. [70], who found similar reflections in gelatin-glycerol films reinforced with inulin and commercial cellulose nanocrystals (CNC). The shift in these peaks, relative to FC, further supported the presence of crystalline CNC or CMFs, which contributed to improved structural organization within the film. Moreover, an increase in peak intensity at ~22.0° was observed, aligning with previous findings where gelatin films reinforced with bamboo-derived nanocellulose and dialdehyde modifications showed similar behavior, corresponding to triclinic and monoclinic diffraction patterns [73].

Additionally, films based on fish gelatin and Satureja Khuzestanica essential oil have been reported to exhibit broad peaks around 20–21°, indicative of semi-crystalline polymeric systems [50]. The enhancement in peak intensity near 22° in the FO formulation supported the hypothesis that CMFs form intermolecular interactions with gelatin chains, promoting the alignment and packing of polymer segments. This resulted in films that were more crystalline and structurally ordered compared to the unreinforced FC films [74].

### 3.7. Morphological Characterization by Scanning Electron Microscopy (SEM)

**Figure 7:**
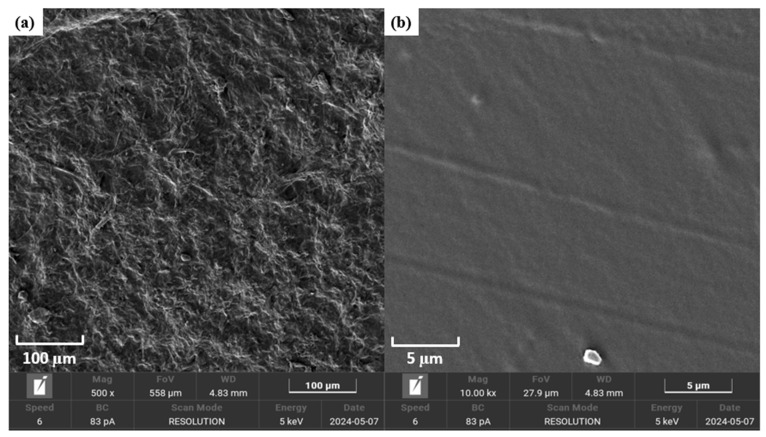
Surface micrographs of films: (**a**) films with CMFs (FO) and (**b**) without CMFs (FC). Note: FO (film optimal); FC (film control).

Figure 7 provides an overview of the surface micrographs of the films with CMFs (FO) and without CMFs (FC).

In this study, the FO formulation displayed a visibly rough surface, which could be attributed to the excessive entanglement of cellulose microfibers (CMFs) within the polymer matrix. The fibrous microstructure disrupted the film’s morphological uniformity, likely due to an uneven distribution of CMFs during the film formation process. This led to discontinuities in the intermolecular bonding network, ultimately compromising the structural integrity and homogeneity of the film surface [25].

Surface roughness is a critical parameter in biopolymer films, as it can either promote or hinder the adsorption of macromolecules onto the film surface, depending on the intended application. Several factors influence surface roughness, including the particle size of the reinforcing material, its degree of dispersion, and the tendency for fiber aggregation, which often occurs during the drying phase of the solvent-casting process. The type of solvent used can also influence the extent of CMF agglomeration. Furthermore, chemical interactions—particularly hydrogen bonding between the gelatin-glycerol matrix and the CMFs—can significantly affect surface topology by altering the internal arrangement of the film’s components [75].

Wang et al. [76], investigating the incorporation of rice husk-derived cellulose in gelatin-based films, reported network-like fiber entanglements indicative of incomplete dissolution of cellulose in the casting solution. This incomplete dispersion resulted in a heterogeneous surface morphology, similar to what was observed in the FO films. Comparable evidence was provided by Sharma et al. [24] and in films produced with bovine gelatin and CNC derived from eucalyptus kraft pulp, where the lack of uniform distribution of nanocellulose disrupted the film structure and increased surface roughness [28].

## 4. Conclusions

This study successfully demonstrated the development and comprehensive characterization of biodegradable gelatin-based films reinforced with cellulose microfibrils (CMFs) derived from cowpea pod skin (CPMC) and corn straw (CSMC). Aligned with the study’s objectives, the incorporation of 1.00% CPMC and 1.00% CSMC into the gelatin–glycerol matrix markedly improved the films’ functional properties, particularly in terms of tensile strength, water vapor permeability, and solubility, when compared to the control formulation without CPMC and CSMC. The developed film exhibited a four-fold increase in mechanical resistance, a 55% reduction in water vapor permeability, and enhanced thermal stability, without compromising its biodegradability, which reached 73% within three days. These improvements were attributed to the formation of strong hydrogen bonding and interfacial interactions between the CMFs and the biopolymer matrix. Therefore, the results validated the feasibility of converting food processing residues into eco-friendly packaging materials.

Future investigations should explore the integration of bioactive compounds to further enhance antimicrobial or antioxidant activity, opening avenues for active packaging applications that align with current trends of sustainable food preservation.

## Figures and Tables

**Table 1:** Design matrix: independent variables X_1_ (corn straw microcellulose, CSMC) and X_2_ (cowpea pod skin microcellulose, CPMC). The response variables: TS (tensile strength), EB (elongation at break), and WVP (water vapor permeability).

Formulations	Independent Variables		Response Variables		
	X_1_ (%)	X_2_ (%)	TS (MPa)	EB (%)	WVP (× 10^−4^ gH_2_O.mm/m^2^.h.mmHg)
1	0.29	0.29	0.84	66.27	4.11
2	0.29	1.71	2.50	23.81	5.85
3	1.71	0.29	3.10	32.17	3.17
4	1.71	1.71	2.51	24.13	4.00
5	0.00	1.00	1.17	49.43	3.03
6	2.00	1.00	4.42	29.12	3.52
7	1.00	0.00	1.48	31.78	2.47
8	1.00	2.00	2.76	27.43	3.83
9	1.00	1.00	2.71	30.66	2.82
10	1.00	1.00	2.73	30.70	2.83
11	1.00	1.00	2.68	30.68	2.80

Note: X_1_ (corn straw microcellulose, CSMC) and X_2_ (cowpea pod skin microcellulose, CPMC); TS (tensile strength); EB (elongation at break); WVP (water vapor permeability).

**Table 2:** Characterization of the films.

Parameters	Results	
	FO	FC
TS (MPa)	2.71 ± 0.02	0.68 ± 0.02
EB (%)	30.68 ± 0.16	143.52 ± 2.10
WVP (×10^−4^ gH_2_O.mm/m^2^ h. mmHg)	2.82 ± 0.01	6.33 ± 0.09
Thickness (mm)	0.16 ± 1.12	0.11 ± 1.27
L*	81.33 ± 0.24	85.34 ± 0.42
a*	0.03 ± 0.02	−0.51 ± 0.02
b*	13.51 ± 0.33	2.63 ± 0.02
ΔE	11.61	--
Opacity	5.69 ± 0.37	1.05 ± 0.35
Solubility (%)	75.82 ± 0.97	89.64 ± 1.80
Degradability (%)		
Day 1	64.43 ± 0.27	100 ± 0.00
Day 2	68.43 ± 0.27	--
Day 3	73.06 ± 0.04	--

Note: FO = Film optimal; FC = Film control; TS (tensile strength); EB (elongation at break); WVP (water vapor permeability); ΔE (total color difference); L* (luminosity), a* (red-green component) and b* (yellow-blue component).

## Data Availability

Data will be made available upon request.
